# Expression Profiling of Coding and Noncoding RNAs in the Endometrium of Patients with Endometriosis

**DOI:** 10.3390/ijms251910581

**Published:** 2024-10-01

**Authors:** Mi Ran Choi, Hye Jin Chang, Jeong-Hyeon Heo, Sun Hyung Yum, Eunae Jo, Miran Kim, Sang-Rae Lee

**Affiliations:** 1 Laboratory Animal Research Center, Ajou University School of Medicine, Suwon 16499, Republic of Korea; mrchoi2007@ajou.ac.kr; 2 Department of Pharmacology, Ajou University School of Medicine, Suwon 16499, Republic of Korea; hjh2g@ajou.ac.kr; 3 Department of Obstetrics and Gynecology, Ajou University School of Medicine, Suwon 16499, Republic of Korea; zzanga-94@hanmail.net (H.J.C.); yumdoc01@naver.com (S.H.Y.); dmsdo_@naver.com (E.J.)

**Keywords:** endometriosis, endometrium, menstrual cycle, long noncoding RNA, neighboring gene

## Abstract

The aim of this study was to identify differentially expressed lncRNAs (DElncRNAs) and mRNAs (DEmRNAs) in the endometrium of individuals with and without endometriosis (EMS) during the proliferative (P) and secretory (S) phases of the menstrual cycle. Tissues were obtained from 18 control (CT; P-phase [pCT], *n* = 8; S-phase [sCT], *n* = 13) and 23 EMS patients (P-phase [pEMS], *n* = 13; S-phase [sEMS], *n* = 12). DElncRNAs and DEmRNAs were analyzed using total RNA-sequencing. In P-phase, expression of *NONHSAG019742.2* and *NONHSAT120701.2* was significantly higher in EMS than control patients, that of while *NONHSAG048398.2* and *NONHSAG016560.2* was lower in EMS patients. In S-phase, expression of *NONHSAT000959.2*, *NONHSAT203423.1*, and *NONHSAG053769.2* was significantly increased in EMS patients, while that of *NONHSAG012105.2* and *NONHSAG020839.2* was lower. In addition, the expression of *HSD11B2*, *THBS1*, *GPX3*, and *SHISA6* was similar to that of neighboring lncRNAs in both P- and S-phases. In contrast, *ELP3* and *NR4A1*, respectively, were up- or downregulated in pEMS tissues. In sEMS, expression of *LAMB3* and *HIF1A* was increased, while expression of *PAM* was reduced. Our findings on lncRNAs and mRNAs encourage not only exploration of the potential clinical applications of lncRNAs and mRNAs as prognostic or diagnostic biomarkers for EMS but also to gain valuable insights into its pathogenesis.

## 1. Introduction

The menstrual cycle consists of two main phases: the proliferative (P-phase) and secretory (S-phase), each regulated by distinct hormonal influences. Estrogen is the dominant hormone during the P-phase, promoting cellular proliferation, angiogenesis, and preparing the endometrium for potential implantation. In cases of endometriosis (EMS), disruptions in estrogen-dependent signaling pathways can lead to abnormal proliferation of ectopic endometrial cells, further aggravating the progression of the disease [1]. On the other hand, the S-phase is primarily driven by progesterone, which typically counterbalances estrogen-driven proliferation and supports the differentiation of endometrial cells. However, in women with EMS, there is often a resistance to progesterone, where endometrial tissue does not respond properly to its effects [2]. This resistance is linked to changes in the expression of progesterone receptors and related signaling pathways, reducing progesterone’s anti-inflammatory and anti-proliferative roles.

EMS is a chronic gynecological disease in which endometrial tissue, which is normally located inside the uterus, grows outside of it. This abnormal tissue may be found on various organs and structures, including the fallopian tubes, ovaries, or peritoneum. Both eutopic and ectopic uterine endometrial tissues respond to hormonal signals during the menstrual cycle; however, ectopic endometrial tissue is not expelled from the body during menstruation, leading to inflammation, scar tissue formation, and potential impact on uterine endometrial tissue. EMS occurs in 10 to 15% of women of reproductive age and causes various symptoms such as pelvic pain, lower or abdominal pain, pain with bowel movements or urination, pain with intercourse, longer and heavier menstrual bleeding, and infertility [3,4,5]. Therefore, a better understanding of the etiology and pathophysiology of EMS is needed to identify biomarkers and to develop more effective strategies to prevent, diagnose, and treat EMS and prevent relapses; such investigations should include genomic assessment.

The human genome is composed of coding and noncoding DNAs that are transcribed to coding and noncoding RNAs, respectively. Coding messenger RNAs (mRNAs) are translated into proteins. Among noncoding RNAs (ncRNAs), there are three main categories based on size: small RNAs (17–25 nucleotides), which include microRNAs (miRNAs); mid-size RNAs (20–200 nucleotides); and long ncRNAs (lncRNAs), consisting of more than 200 nucleotides [6]. The novel high-throughput RNA-sequencing (RNA-Seq) technology has revolutionized transcriptomics, allowing discovery of known and unknown transcripts and noncoding RNAs [7,8,9,10], including rapidly identifying the existence and function of several novel lncRNAs [11]. As a result, lncRNAs are emerging as promising therapeutic targets in numerous diseases including EMS, neurodegenerative diseases, and cancer due to newly identified roles in various key processes, such as epigenetic and transcriptional modifications and cell signaling pathways [12,13,14].

A microarray study predicted that lncRNAs participate in crucial processes associated with the pathogenesis of EMS, including immune responses, estrogen production, tissue adhesion, and angiogenesis [15]. Additional high-throughput studies have found differential expression of multiple immune-related lncRNAs in ectopic and eutopic endometrial samples, which included lncRNAs that modulate signaling pathways such as PI3-K-AKT and MAPK [16,17]. Another study investigated differential expression of lncRNAs, circRNAs, and mRNAs in eutopic and ectopic endometrial tissue using next generation sequencing (NGS) technology and identified endogenous networks among them [18]. Earlier reports showed lncRNAs to be involved in various pathological mechanisms by regulating mRNA stability and mRNA translation [19,20]. Nevertheless, there have been few studies investigating lncRNA expression and the correlations between lncRNAs and mRNAs in EMS tissue.

The endometrial transition from the P-phase to S-phase during the menstrual cycle entails maturation of secretory glands and adjustment of uterine fluid composition through secretion of factors that govern implantation and embryo development [21]. We hypothesized that the patterns of lncRNA and mRNA expression in endometrial tissue during the menstrual cycle would differ between normal control (CT) and EMS patients. Thus, we aimed to analyze the expression of lncRNAs and mRNAs in eutopic tissue and to identify their networks. To accomplish this, we performed high-throughput profiling of lncRNAs and mRNAs in eutopic endometrial tissues from CT and EMS patients using total RNA-Seq and functional annotation of differentially expressed mRNAs. In addition, we validated genes selected from total RNA-Seq using qRT-PCR and identified some candidate genes as EMS-related biomarkers. Our findings provide new data regarding the expression of lncRNAs and mRNAs and their correlated expression in eutopic endometrium during the menstrual cycle.

## 2. Results

### 2.1. Identification of DElncRNAs in the Control and EMS Groups during the Menstrual Cycle

**Figure 1:**
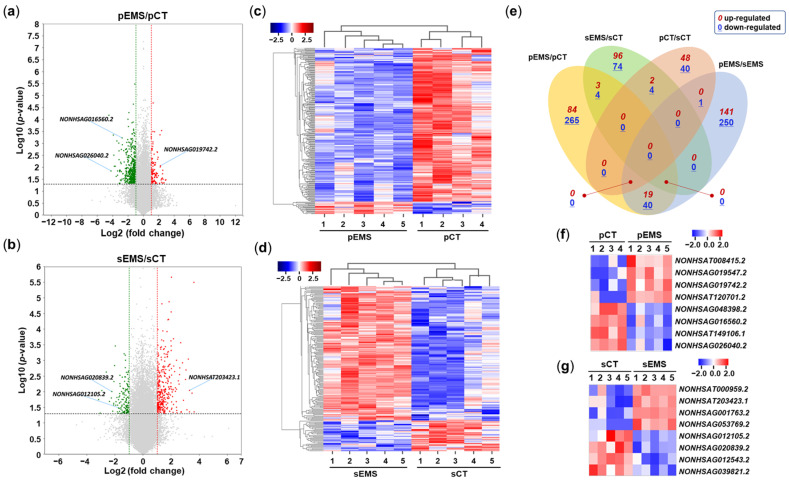
Differential expression of long noncoding RNA (lncRNA) in the control (CT) and endometriosis (EMS) groups. (**a**) Volcano plot of differentially expressed lncRNAs (DElncRNAs) in the proliferative-phase CT (pCT) and EMS (pEMS) groups. (**b**) Volcano plot of DElncRNAs in the secretory-phase CT (sCT) and EMS (sEMS) groups. (**c**) Heatmap generated from hierarchical clustering analysis of DElncRNAs in the pCT and pEMS groups. (**d**) Heatmap generated from hierarchical clustering analysis of DElncRNAs in the sCT and sEMS groups. (**e**) Venn diagram showing overlap of lncRNA expression among the pCT, pEMS, sCT, and sEMS groups. (**f**,**g**) Expression of select lncRNAs in the CT and EMS groups as assessed using qRT-PCR.

We used total RNA-Seq to perform high-throughput transcriptomic profiling of eutopic endometrial tissues in the P- and S-phases of the menstrual cycle in the CT and EMS groups. After selecting a large amount of data for lncRNAs from the CT and EMS groups using log2 transformation (fold change cutoff of 2), volcano plots of the differentially expressed lncRNAs (DElncRNAs) (Figure 1a,b) and heatmaps showing hierarchical clustering of DElncRNAs were generated using ExDEGA (Excel-based differentially expressed gene analysis; Ebiogen Inc., Seoul, Republic of Korea). When comparing the expression patterns between the CT and EMS groups, 221 lncRNAs of the 240 DElncRNAs identified in P-phase endometrium samples were downregulated in the pEMS group compared with the pCT group, while 154 lncRNAs of the 189 DElncRNAs in S-phase samples were upregulated in the sEMS group compared with the sCT group (Figure 1a,b and Table S1). As shown in Figure 1c,d, the expression of endometrial lncRNAs was correlated among samples within each group. The Venn diagrams presented in Figure 1e show up/downregulated lncRNAs among comparison pairs: the largest number of DElncRNAs was identified between the pEMS and sEMS groups, and the second largest was for comparison of the pEMS and pCT groups.

### 2.2. Identification of DEmRNAs between NC and EMS Groups during the Menstrual Cycle

**Figure 2:**
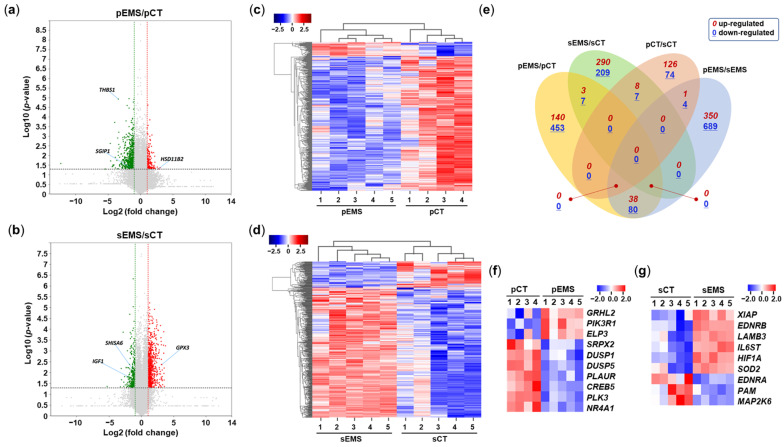
Differential expression of mRNAs in the control (CT) and endometriosis (EMS) groups. (**a**) Volcano plot of differentially expressed mRNAs (DEmRNAs) in the proliferative-phase CT (pCT) and EMS (pEMS) groups. (**b**) Volcano plot of DEmRNAs in the secretory-phase CT (sCT) and EMS (sEMS) groups. (**c**) Heatmap generated from hierarchical clustering analysis of DEmRNAs in the pCT and pEMS groups. (**d**) Heatmap generated from hierarchical clustering analysis of DEmRNAs in the sCT and sEMS groups. (**e**) Venn diagram showing overlap of mRNAs among the pCT, pEMS, sCT, and sEMS groups. (**f**,**g**) Expression of select DEmRNAs in the CT and EMS groups as assessed using qRT-PCR.

After selecting differentially expressed mRNAs (DEmRNAs) with fold-change values ≥ 2, volcano plots of the DEmRNAs and heatmaps showing hierarchical clustering of them were created using ExDEGA (Ebiogen Inc., Seoul, Republic of Korea). When comparing the expression patterns of the NC and EMS groups, 365 mRNAs of the 407 DEmRNAs in P-phase endometrial samples were downregulated in the pEMS group compared with the pCT group, while 431 mRNAs among the 520 DEmRNAs in S-phase samples were upregulated in the sEMS group compared with the sCT group (Figure 2a,b). The expression of endometrial mRNAs was correlated among samples within groups (Figure 2c,d). When the numbers of up/downregulated mRNAs or overlapped mRNAs among comparison pairs were presented as Venn diagrams (Figure 2e), the four comparison pairs showed many DEmRNAs but little overlap of mRNAs.

### 2.3. Functional Annotation and Pathway Networks of mRNAs

**Figure 3:**
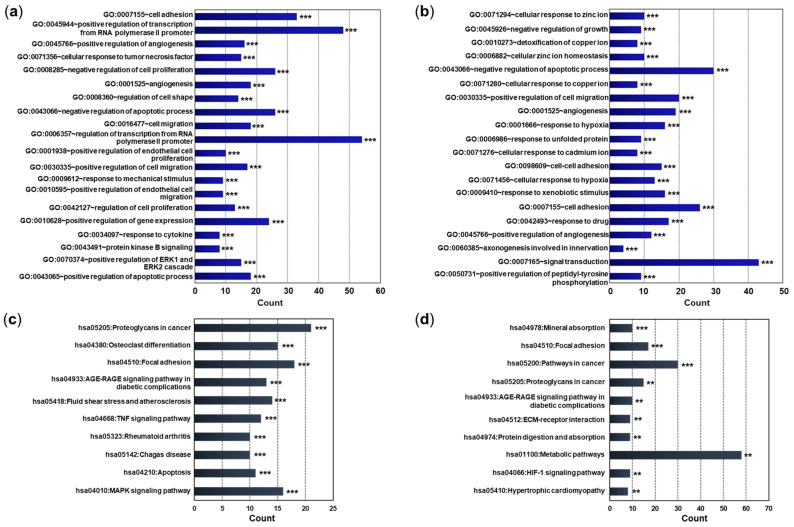
Classification of differentially expressed mRNAs (DEmRNAs) between the control (CT) and endometriosis (EMS) groups as assessed using gene ontology (GO) and Kyoto Encyclopedia of Genes and Genomics (KEGG) PATHWAY enrichment. (**a**) Top 20 enriched GO terms in the biological process (BP) category among DEmRNAs in the pEMS group compared with the pCT group. (**b**) Top 20 enriched GO terms in the BP category among DEmRNAs in the sEMS group compared to the sCT group. (**c**) Top 10 enriched KEGG pathways in the pEMS group compared with the pCT group. (**d**) Top 10 enriched KEGG pathways in the sEMS group compared with the sCT group. The X-axis represents the number of genes. ** *p* < 0.001, *** *p* < 0.0001.

After performing GO enrichment and functional annotation analysis using DAVID bioinformatics resources (https://david.ncifcrf.gov (accessed on 24 May 2024)), we identified three GO categories (biological process [BP], cellular component [CP], and molecular function [MF]) and Kyoto Encyclopedia of Genes and Genomics (KEGG) pathways related to DEmRNAs. As a result of enrichment of the top 20 GO terms that satisfied the adjusted *p*-values in the BP category, genes related to cell adhesion, angiogenesis, and negative regulation of apoptotic process were differentially expressed in the pEMS group compared with the pCT group (Figure 3a and Table S2). On the other hand, genes related to cellular response to zinc ion, negative regulation of apoptotic process, and response to hypoxia were differentially expressed in the sEMS group compared with the sCT group (Figure 3b and Table S2). In addition, genes related to apoptotic processes and wound healing were differentially expressed in the pCT and sCT groups, while genes related to angiogenesis and cell adhesion were differentially expressed in the pEMS and sEMS groups (Figure S1a,b). The KEGG pathway enrichment analyses indicated that genes related to proteoglycans in cancer, osteoclast differentiation, and focal adhesion were differentially expressed in the pEMS group compared with the pCT group, while genes related to mineral absorption, focal adhesion, and pathways in cancer were differentially expressed in the sEMS group compared with the sCT group (Figure 3c,d and Table S2). When comparing the pCT and sCT groups, genes related to mineral absorption were differentially expressed; genes related to focal adhesion were differentially expressed between pEMS and sEMS groups (Figure S1c,d).

### 2.4. Pathway Network Identification of DEmRNAs

**Figure 4:**
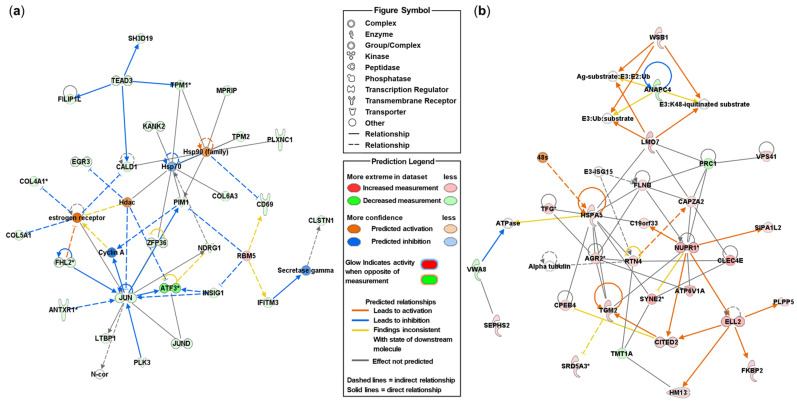
Top networks of differentially expressed mRNAs (DEmRNAs) in the control (CT) and endometriosis (EMS) groups, as identified using the Ingenuity Pathway Analysis (IPA) software (version 122103623; QIAGEN, Hilden, Germany). (**a**) The top network of DEmRNAs in the pEMS group compared with the pCT group. (**b**) The top network of DEmRNAs in the sEMS group compared with the sCT group. An asterisk indicates that multiple identifiers in the dataset file map to a single gene or chemical in the Global Molecular Network.

To better understand the biological mechanisms and cellular pathways in endometrial tissue, DEmRNAs were analyzed using Ingenuity Pathway Analysis (IPA) software (version 122103623; QIAGEN, Hilden, Germany). When comparing DEmRNAs between the pCT and pEMS groups, 20 networks were identified (Table S3). In addition, 28 focus molecules were included in the top-ranked network (IPA score: 41) and were associated with cardiovascular disease, cell death and survival, and connective tissue disorders (Figure 4a and Table S3). When comparing the sCT and sEMS groups, 20 networks were identified; 29 focus molecules were included in the top-ranked network (IPA score: 42) and were associated with organismal injury and abnormalities, organismal survival, and cellular movement (Figure 4b and Table S3). When comparing the pCT and sCT groups, 13 networks were identified; 24 focus molecules were included in the top-ranked network (IPA score: 43) and were associated with neurological disease, organismal injury and abnormalities, and cardiovascular system development and function (Figure S2a and Table S3). When comparing the pEMS and sEMS groups (Figure S2b), 31 focus molecules were included in the top-ranked network (IPA score: 40). In addition, when we selected 30 canonical pathways involved in the CT and EMS groups by comparative analysis based on z-score, Pulmonary Fibrosis Idiopathic Signaling Pathway was downregulated in both pEMS and sEMS groups (Figure S3).

### 2.5. Validation of lncRNAs and Neighboring Genes Based on qRT-PCR

**Figure 5:**
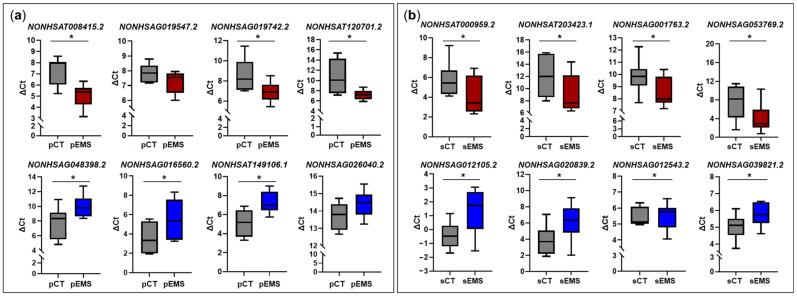
Validation of differentially expressed long noncoding RNAs (DElncRNAs) during the menstrual cycle of the control (CT) and endometriosis (EMS) groups. Select DElncRNAs were validated using qRT-PCR. (**a**) DElncRNAs in the EMS group (*n* = 13) compared with the CT group (*n* = 8) during the proliferative phase of the menstrual cycle. (**b**) DElncRNAs in the EMS group (*n* = 12) compared with the CT group (*n* = 13) during the secretory phase of the menstrual cycle. * *p* < 0.05.

To validate the expression of lncRNAs identified in the total RNA-Seq analyses, we selected 16 lncRNAs and analyzed them using qRT-PCR. In P-phase endometrial samples, three lncRNAs (*NONHSAT008415.2*, *NONHSAG019742.2*, and *NONHSAT120701.2*) were significantly upregulated, and three (*NONHSAG048398.2*, *NONHSAG016560.2*, and *NONHSAT14106.1*) were significantly downregulated in the pEMS group compared with the pCT group (Figure 5a). In addition, *NONHSAG019547.2* and *NONHSAG026040.2* showed a tendency to be up- or downregulated, respectively, in the pEMS group. On the other hand, in S-phase samples, four lncRNAs (*NONHSAT000959.2*, *NONHSAT203423.1*, *NONHSAG001763.2*, and *NONHSAG053769.2*) were significantly upregulated and three (*NONHSAG012105.2*, *NONHSAG020839.2*, and *NONHSAG039821.2*) were significantly downregulated in the sEMS group compared with the sCT group (Figure 5b). All validated lncRNAs except *NONHSAG012543.2* showed similar expression patterns to those observed in the total RNA-Seq analyses (Figure 1f,g).

**Figure 6:**
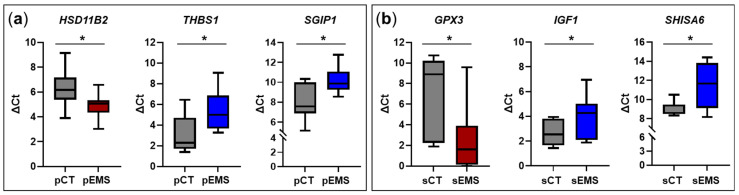
Validation of the differential expression of genes near select validated lncRNAs in the control (CT) and endometriosis (EMS) groups during the menstrual cycle. (**a**) Changes in the expression of neighboring genes in the EMS group (*n* = 13) compared with the CT group (*n* = 8) during the proliferative phase of the menstrual cycle. (**b**) Changes in the expression of neighboring genes in the EMS group (*n* = 12) compared with the CT group (*n* = 13) during the secretory phase of the menstrual cycle. * *p* < 0.05.

In light of reports that lncRNAs *cis*-regulate the expression of neighboring genes [22,23,24], we evaluated neighboring genes of validated lncRNAs. In P-phase, *HSD11B2*, a neighboring gene of *NONHSAG019742.2*, was significantly upregulated like the expression of *NONHSAG019742.2* (Figure 1a, Figure 2a and Figure 6a). In contrast, expression of *THBS1* and *SGIP1,* which are neighboring genes of *NONHSAG016560.2* and *NONHSAG026040.2*, respectively, was significantly downregulated similar to their related lncRNAs. In S-phase endometrial samples, *GPX3*, a neighboring gene of *NONHSAT203423.1*, was significantly upregulated, similar to the expression of *NONHSAT203423.1* (Figure 1b, Figure 2b and Figure 6b). On the other hand, *IGF1* and *SHISA6* that are neighboring genes of *NONHSAG012105.2* and *NONHSAG020839.2*, respectively, were significantly downregulated, similar to the related lncRNAs.

### 2.6. Validation of mRNAs Based on qRT-PCR

**Figure 7:**
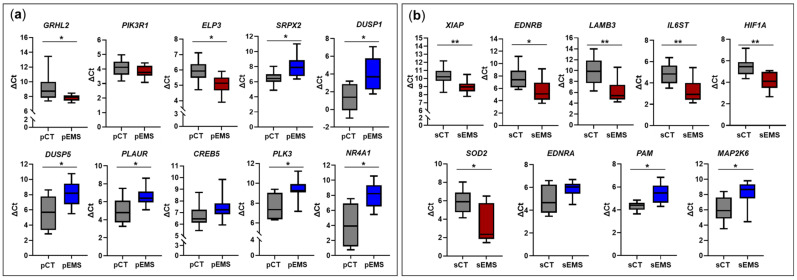
Validation of differentially expressed mRNAs (DEmRNAs) in the control (CT) and endometriosis (EMS) groups during the menstrual cycle. (**a**) Changes in the expression of select mRNAs in the EMS group (*n* = 13) compared with the CT group (*n* = 8) during the proliferative phase of the menstrual cycle. (**b**) Changes in the expression of some mRNAs in the EMS group (*n* = 12) compared with the CT group (*n* = 13) during the secretory phase of the menstrual cycle. * *p* < 0.05, ** *p* < 0.01.

Based on the total RNA-Seq results (Table S4), we selected ten and nine DEmRNAs for the P- and S-phases, respectively, and validated them using qRT-PCR. The sample number used to validate mRNA expression changes was the same as that used to validate lncRNA expression changes. In the P-phase endometrial samples, genes related to cell adhesion (*GRHL2*) and positive regulation of cell migration (*ELP3*) were significantly upregulated in the pEMS group compared with the pCT group, while genes related to angiogenesis (*SRPX2*), the MAPK signaling pathway (*DUSP1* and *DUSP5*), proteoglycans in cancer (*PLAUR*), negative regulation of apoptotic process (*PLK3*), and positive regulation of endothelial cell proliferation (*NR4A1*) were significantly downregulated in the pEMS group compared with the pCT group (Figure 7a). On the other hand, in the S-phase samples, genes related to negative regulation of apoptotic processes (*XIAP*, *EDNRB*, and *SOD2*) and pathways in cancer (*LAMB3*, *IL6ST*, and *HIF1A*) were significantly upregulated in the sEMS group compared with the sCT group, while genes related to responses to hypoxia (*PAM*) and the Rap1 signaling pathway (*MAP2K6*) were significantly downregulated in the sEMS group compared with the sCT group (Figure 7b). Based on these results, all validated mRNAs showed similar expression patterns to those observed in the total RNA-Seq analyses (Figure 2f,g and Table S4).

## 3. Discussion

Given that the human endometrium is a highly dynamic tissue that experiences a variety of molecular and cellular changes throughout the menstrual cycle and the division between the P- and S-phases is critical in understanding the molecular mechanisms of EMS, transcriptomic studies can provide insights into the complex mechanisms driving EMS. Recently, lncRNAs and mRNAs have been postulated to modulate a variety of processes involved in the pathogenesis of EMS, and lncRNAs, in particular, have emerged as possible biomarkers of EMS. Therefore, we profiled differentially expressed lncRNAs and mRNAs in both P- and S-phase endometrial samples obtained from CT and EMS patients.

In the current study, P-phase endometrial tissues from EMS patients showed changes in the expression of genes related to cell adhesion, positive regulation of angiogenesis, cellular response to tumor necrosis factor, and positive regulation of endothelial cell proliferation. Previous studies have reported that endometrium in EMS patients exhibits increased adhesion and proliferative capacity, and that the development of EMS is associated with proangiogenic processes [13,25]. However, these studies did not consider the phase of the menstrual cycle. When assessing the S-phase, we found altered expression of genes involved in cellular response to zinc ion, negative regulation of growth, negative regulation of apoptotic process, angiogenesis, and response to hypoxia. Metal ions such as zinc and copper have been shown to perform various physiological functions, including enzymatic activation, DNA synthesis and repair, anti-oxidative function, hormonal and cellular signaling, and immunomodulation in the S-phase of the menstrual cycle [26,27]. Given that the fertilized egg implants in the endometrium during the S-phase, assessing the expression of genes related to the regulation of these metal ions in the endometrial tissues of EMS patients is expected to be helpful in understanding infertility as well as the pathophysiology of EMS. As the endometrium transitions from the P-phase to the S-phase, with maturation of secretory glands and thickening of the endometrium, there are multiple changes in gene expression, and it is reasonable that some gene functions between P- and S-phases differ. These differences in gene function suggest that the menstrual cycle be considered when searching for biomarkers targeting EMS.

Even though lncRNAs do not code for proteins, they control mRNA expression both transcriptionally and post-transcriptionally. Various mechanisms are responsible for lncRNA function. A well-known example is lncRNAs functioning as competing endogenous RNA (ceRNA) to suppress miRNA and manage target mRNA [28]. Additionally, lncRNA can either *cis*-regulate the expression of neighboring genes or *trans*-regulate the expression of distant genes [29]. A growing body of evidence suggests that lncRNA plays a crucial role in endometriosis, with dysregulated lncRNAs showing potential for use as diagnostic biomarkers or targets for therapy. In this study, many lncRNAs were up- or downregulated in tissue samples from patients with EMS compared to CT patients during both the P- and S-phases. Among DElncRNAs in P-phase tissues from EMS patients, *NONHSAG019742.2* was significantly upregulated. Recently, it has been reported that lncRNA was downregulated in the plasma of patients with dilated cardiomyopathy (DCM) [30], as were other circulating lncRNAs. These findings suggest their association with cardiac function and their potential as prognostic biomarkers for DCM. On the other hand, Cai et al. identified that *NONHSAG026040.2*, also known as *lnc-FOSB-1-1*, was downregulated in the blood of patients with systemic lupus erythematous (SLE), and that the decrease was associated with greater risk of renal damage [31]. The expression of *NONHSAG026040.2* was also downregulated in the P-phase tissues of EMS patients in this study. The results in both SLE and EMS patients indicate that changes in the expression of *NONHSAG026040.2* affect the development of some diseases, and that the gene may have value as a diagnostic biomarker for these diseases.

In the present study, when assessing S-phase tissues, *NONHSAG053769.2* was upregulated in the EMS group compared with the CT group. In luteinizing hormone (LH) + 7 (receptive) human endometrium, lncRNA was highly expressed during the luteal phase of the ovarian cycle, coinciding with the S-phase of the menstrual cycle [30]. However, there was no difference in lncRNA expression between patients with or without recurrent implantation failure (RIF), suggesting that the lncRNA does not affect implantation failure [32]. Taken together, these findings indicate that *NONHSAG053769.2*, which is highly expressed during the S-phase, influences the development of endometriosis but not RIF; however, such research is still in the early stages.

Based on reports that lncRNA positively cis-regulates the expression of neighboring genes [22,23], we analyzed the neighboring genes *HSD11B2*, *THBS1*, *SGIP1*, *GPX3*, *IGF1*, and *SHISA6* of *NONHSAG019742.2*, *NONHSAG016560.2*, *NONHSAG026040.2*, *NONHSAT203423.1*, *NONHSAG012105.2*, and *NONHSAG020839.2*, respectively. These neighboring genes showed the same expression pattern as lncRNAs. In P-phase tissues, *HSD11B2*, located near *NONHSAG019742.2*, was significantly upregulated in EMS group compared to the CT group; in contrast, in S-phase tissues, the gene was significantly downregulated in EMS group in total RNA-Seq. Hydroxysteroid 11-beta dehydrogenase 2, encoded by *HSD11B2*, deactivates cortisol by catalyzing its transformation into cortisone [33]. Patients with EMS exhibit abnormalities in the synthesis, degradation, and binding of steroid hormones and in the expression of genes or proteins involved in steroid hormone metabolism and regulation [34]. In the study by the Monsivais team [34], who analyzed the transcriptome in P-phase eutopic and ectopic (ovarian) endometrial tissue obtained from the same EMS patient (*n* = 8), *HSD11B2* expression was significantly lower in the eutopic endometrium than in the ectopic endometrium, contrary to our findings. The opposing pattern of *HSD11B2* expression in this study and our study may have been due to Monsivais et al. having obtained both normal and EMS tissues from the same patients, whereas we obtained normal and EMS tissues from NC and EMS patients, respectively. In the present study, *SHISA6* was shown to be located near *NONHSAG020839.2* and both transcripts were downregulated in S-phase tissues from EMS patients. It has been reported that SHISA6 regulates self-renewal and differentiation of spermatogonia stem cells through Wnt/beta-catenin signaling, implying that SHISA6 may be related to fertility [35,36]. These findings indicate that *NONHSAG020839.2* in S-phase endometrium may also play a role in Wnt/beta-catenin signaling either directly or indirectly. Thus, we postulate the effects of as-yet unidentified lncRNAs on the development of EMS through the actions of neighboring genes.

In the present study, *ELP3* involved in positive regulation of cell migration in GO enrichment was significantly upregulated in P-phase endometrial samples from EMS patients. The ELP3 protein enhances tumorigenesis by stabilizing c-myc, and ELP3 and c-Myc are overexpressed in hepatocellular carcinoma and colorectal cancer [37]. In contrast, the ELP3 protein exhibited high expression in endometrioid adenocarcinoma, and its expression was inversely correlated with progression of the disease [38]. In a microarray transcriptomic study of endometrial tissues obtained from normal controls and IVF patients, *ELP3* was upregulated 2.7-fold in IVF patients, implying that its expression is negatively correlated with fertility [39]. Taken together, these findings indicate ELP3 to be associated with endometrial tissue function and the occurrence of endometrium-related diseases, but the mechanism remains to be elucidated.

An orphan nuclear receptor, NR4A1, is rapidly activated in response to diverse stressors, serving as an immediate-early gene. It plays a pivotal role in cancer cell functions including cell cycle progression, survival, migration, and invasion in diverse solid tumors such as melanoma, pancreatic, lung, and ovarian cancers [40,41]. In the present study, *NR4A1* was significantly downregulated in the endometrium of EMS patients during both the P-phase (in both the total RNA-Seq and qRT-PCR analyses) and S-phase (in the total RNA-Seq analyses). In a study assessing the phosphorylation of NR4A1 (pNR4A1) in ectopic (ovarian) endometrial tissues of EMS patients and P-phase eutopic endometrial tissues of normal controls, ovarian endometriotic tissues had higher pNR4A1 expression than normal endometrial tissues [41]. However, since the specific phase of the menstrual cycle was not identified in the tissues of EMS patients, unlike the P-phase tissues of normal controls, further evaluation is necessary to determine whether the same results are obtained when analyzing autopsy tissues from EMS patients and normal controls during the same phase of menstrual cycle. On the other hand, a study found *NR4A1* expression in S-phase endometrial tissue to be lower in patients with EMS or primary infertility than in normal controls [42], consistent with the findings of our study. Thus, the expression patterns of *NR4A1* appear to differ between endometriosis and endometrial or ovarian cancers, suggesting different regulatory mechanisms. In addition, it is thought that altered expression of *NR4A1* in reproductive-age women with EMS may be associated with infertility.

In this study, when validating select genes for which the expression was altered in S-phase endometria tissues from EMS patients (*LAMB3*, *IL6ST*, and *HIF1A* belonging to pathways in cancer), the significant increase expression of all of these genes was consistent with the results of the total RNA-Seq analyses. The LAMB3 protein is a major component of the extracellular matrix and basal membrane and promotes cell migration and tumorigenicity in SCID mice and lung adenocarcinoma [43,44]. The expression of both LAMB3 mRNA and protein has been reported to be greater in ovarian cancer than in normal ovarian tissue, based on studies that examined correlations between laminin expression and prognosis in ovarian cancer using several open sources including cBioPortal and ONCOMINE [45]. Another study found *LAMB3* mRNA expression to be greater in endometrial cancer than in ovarian cancer [46]. A hypoxic environment induces EMS, endometrioid endometrial cancer, and ovarian endometrial carcinoma, and these diseases induce upregulation of HIF1A in response to hypoxia [47,48,49]. Increased expression of HIF1A triggers various physiological responses, including glycolysis, to mitigate oxygen deficiency and promote angiogenesis. The serum concentration of HIF1A was reported to be greater in patients with EMS than in controls, and the expression of HIF1A increased in both serum and tissues as the severity of EMS increased [50]. Similarly, we found that *HIF1A* mRNA expression in endometrial tissues was greater in EMS patients than in controls, confirming that endometrial hypoxia occurs in EMS, resulting in upregulation of HIF1A. Considering the above-noted findings, our observation that the expression of genes such as *LAMB3* and *HIF1A* increased in EMS patients suggests that early treatment of EMS may be very important to prevent progression to endometrial cancer.

In summary, we profiled DElncRNAs and DEmRNAs in EMS patients during the P- and S-phases of the menstrual cycle. In P-phase endometrial tissues, the expression of both *NONHSAG019742.2* lncRNA and its neighboring gene, *HSD11B2*, increased in EMS patients, while the expression of both *NONHSAG016560.2* lncRNA and its neighboring gene, *THBS1*, decreased in EMS patients. In S-phase endometrial tissues, the expression of both *NONHSAT203423.1* lncRNA and its neighboring gene, *GPX3*, increased in EMS patients, while the expression of both *NONHSAG020839.2* lncRNA and its neighboring gene, *SHISA6*, decreased in EMS patients. In addition, *ELP3* and *NR4A1* that positively regulate cell migration and endothelial cell proliferation, respectively, were up- or downregulated in P-phase obtained from EMS patients. In S-phase tissues, the expression of *LAMB3* and *HIF1A*, associated with pathways in cancer, increased in EMS patients, while the expression of *PAM,* related to responses to hypoxia, was reduced.

While we identified significant DElncRNAs and DEmRNAs in EMS patients during the P- and S- phases of the menstrual cycle, this study has certain limitations. First, the relatively small sample size may limit the generalizability of the findings. A larger cohort could have provided more statistically robust and reliable data on the differences in lncRNA and mRNA expression between EMS patients and controls. Second, there is potential for technical variability, particularly in RNA extraction and qRT-PCR. To mitigate these issues, we followed standardized protocols for RNA extraction across all samples, and qRT-PCR reactions were performed in triplicate to minimize variability in amplification efficiency. Despite these measures, residual variability from pipetting or reaction conditions may still have affected the results. Nonetheless, this study is, to the best of our knowledge, the first study to identify changes in the expression of both lncRNAs and mRNAs depending on the phase of the menstrual cycle in women with EMS. Given that EMS, an estrogen-dependent disease, encounters different endocrine profiles during the P- and S-phases, our results suggest the need for further exploration of the clinical applications of DElncRNAs and DEmRNAs as prognostic or diagnostic biomarkers for EMS and may to lead to valuable insights into the pathogenesis of EMS. Additionally, further studies will be necessary to investigate the specific roles of these potential biomarker candidates, providing a more comprehensive understanding of the molecular mechanisms underlying EMS pathogenesis.

## 4. Materials and Methods

### 4.1. Sample Collection

The study recruited premenopausal women (aged 27–45) undergoing surgery for conditions such as EMS and benign gynecological disorders (e.g., uterine fibroids, endometrial polyps, benign ovarian tumors) at Ajou University Hospital. After explaining the study protocol and obtaining informed consent, all participants provided written consent for the study. Women taking hormonal medications that could affect endometrial conditions or having a malignant disease were excluded. Participants were divided into two groups: a control group (CT group) confirmed to have no pelvic or ovarian EMS at the time of surgery, and an EMS group confirmed to have pelvic EMS or endometriotic lesions on histopathological examination. Eutopic endometrial tissues were collected via pipelle biopsy or hysteroscopy during the surgical procedure. Samples were analyzed based on the menstrual cycle phase at the time of collection, categorized into P- and S-phases.

Three samples (CT group, *n* = 1; EMS group, *n* = 2) were provided by the Biobank of AJOU University Hospital (Suwon-si, Republic of Korea), a member of Korea Biobank Network. After excluding unsuitable subjects as described above, the study utilized endometrial tissues from a total of 43 participants (the CT group, *n* = 18; the EMS group, *n* = 25).

### 4.2. Library Preparation and Sequencing

Total RNA was extracted from eutopic endometrial tissues obtained from patients in the P-phase (CT group [proliferative control, pCT], *n* = 4; EMS group [proliferative EMS, pEMS], *n* = 5) and S-phase (CT group [secretory control, sCT], *n* = 5; EMS group [secretory EMS, sEMS], *n* = 5) of their menstrual cycle using TRIzol™ reagent (Thermo Fisher Scientific, Waltham, MA, USA). The quality of the total RNA recovered was assessed using an Agilent 4200 TapeStation system (Agilent Technologies, Santa Clara, CA, USA), and the RNA integrity number (RIN) was higher than 7.8 in all samples. After removing undesired ribosomal RNA (rRNA) using a RiboCop rRNA Depletion kit (LEXOGEN Inc., Vienna, Austria), libraries were constructed using an NEBNext Ultra II Directional RNA Library Prep kit (New England BioLabs Inc., Ipswich, MA, USA) according to the manufacturer’s instructions. The enrichment of the libraries was carried out using PCR, and the libraries were checked using Agilent High Sensitivity DNA kit (Agilent Technologies, Santa Clara, CA, USA) on the Agilent 2100 bioanalyzer (Agilent Technologies, Santa Clara, CA, USA) to evaluate the mean fragment size. The constructed libraries were 101-bp paired-end sequenced using a NovaSeq 6000 system (Illumina, San Diego, CA, USA).

### 4.3. Differerential Gene Expression Analysis and Functional Annotation

Quality-control assessment of raw sequencing data was performed using FastQC [51]. Adapter contamination and low-quality reads (<Q20) were removed using FASTX_Trimmer [52] and BBMap [53]. The trimmed reads were mapped to the reference genome using TopHat [54]. The expression of genes, isoforms, and lncRNAs was estimated using Fragments Per kb per Million reads (FPKM) values by Cufflinks [55]. The FPKM values were normalized based on the quantile normalization method using edgeR. To facilitate the log2 transformation, a value of 1 was added to each FPKM value of the transcriptomes. The *p*-values obtained from edgeR were adjusted using the Benjamini–Hochberg method to determine the false discovery rate (FDR) and find out DEmRNAs and DElncRNAs. No batch correction was performed, as no significant batch effect was observed. All mRNAs and ncRNAs for which the fold change in expression was ≥ 2.0 in at least one of the comparison pairs were selected. For DEmRNA and DElncRNA sets, the volcano plot and hierarchical clustering analyses were performed using ExDEGA (Ebiogen Inc., Seoul, Republic of Korea). Gene ontology (GO) enrichment and functional annotation analysis of DEmRNAs were performed using DAVID Bioinformatics Resources “https://david.ncifcrf.gov (accessed on 24 May 2024)”. Additional analyses of the biological responses and various canonical pathways associated with DEmRNAs were performed using IPA software (version 122103623; QIAGEN, Hilden, Germany). The IPA identified cellular networks associated with DEmRNAs based on previously known associations between genes or proteins.

### 4.4. qRT-PCR Analysis

We performed qRT-PCR to validate mRNAs and lncRNAs differentially expressed in total RNA-Seq assays. The number of samples per group was increased for the qRT-PCR assay as follows: pCT (*n* = 8), pEMS (*n* = 13), sCT (*n* = 10), and sEMS (*n* = 12). Total RNA was extracted from tissues using TRIzol™ reagent (Thermo Fisher Scientific, Waltham, MA, USA) and reverse transcribed into cDNA using PrimeScript^TM^ Reverse Transcriptase (Takara, Shiga, Japan) according to the manufacturer’s instructions. Details of the qRT-PCR method have been described previously [56]. The expression of the lncRNAs and mRNAs in each sample was normalized to that of *U6* and *GAPDH*, respectively. The relative expression differences between the NC and EMS groups were calculated using the 2^−ΔΔCT^ method. The housekeeping gene used to analyze the expression of lncRNA was *U6*, and that used to analyze the expression level of mRNA was *GAPDH*. The expression of each gene was quantified as a threshold cycle (Ct) value, and the larger was the Ct value, the lower was the gene expression. The ΔCt value was obtained by subtracting the Ct value of the housekeeping gene from the Ct value of the target gene. Therefore, the larger the ΔCt value was in the sample, the lower the expression of the target gene was. The primers used for amplification of candidate genes are presented in Table S5.

### 4.5. Statistical Analyses

All statistical analyses were conducted using GraphPad Prism 10 software (San Diego, CA, USA). Differences between the CT and EMS groups with respect to the qRT-PCR results were analyzed using unpaired *t*-tests and Mann–Whitney *U* tests for normally distributed variables and non-parametric variables, respectively. All data were expressed as the mean ± standard error of the mean (SEM). A *p*-value < 0.05 was considered statistically significant.

## Data Availability

The datasets generated and analyzed in this study are available from the corresponding author upon reasonable request.

## Supplementary Materials

The following supporting information can be downloaded at: https://www.mdpi.com/article/10.3390/ijms251910581/s1.
