# Supplementary material for: Expression Profiling of Coding and Noncoding RNAs in the Endometrium of Patients with Endometriosis
Source: Int J Mol Sci. 2024 Oct 1;25(19):10581. doi: 10.3390/ijms251910581 (PMC11476965; doi:10.3390/ijms251910581)
Supplement: Supplementary file 1 [file ijms-25-10581-s001.zip › Figure S1.pptx]

## Slide 1
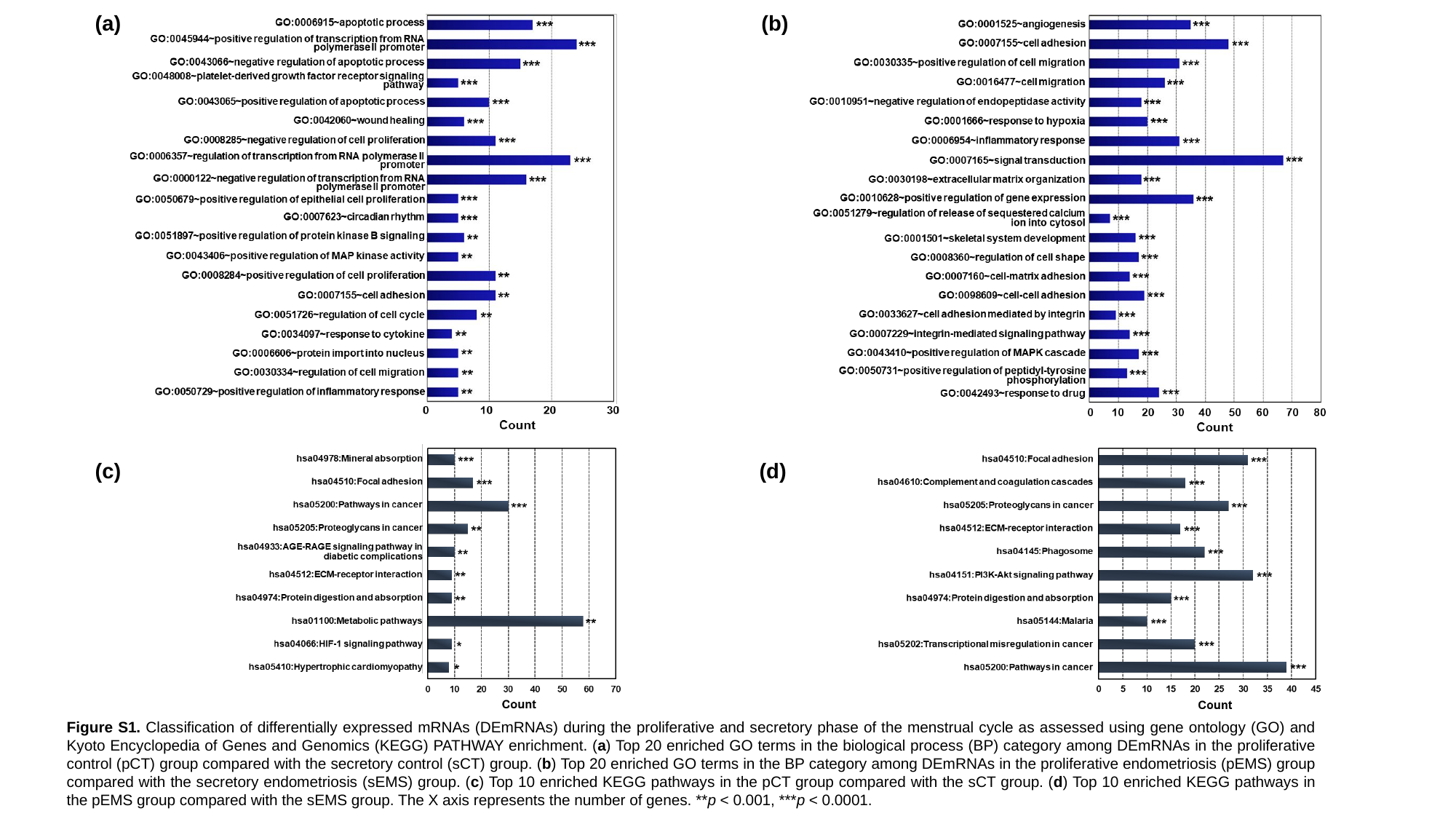

(a)
(b)
(c)
(d)
Figure S1. Classification of differentially expressed mRNAs (DEmRNAs) during the proliferative and secretory phase of the menstrual cycle as assessed using gene ontology (GO) and Kyoto Encyclopedia of Genes and Genomics (KEGG) PATHWAY enrichment. (a) Top 20 enriched GO terms in the biological process (BP) category among DEmRNAs in the proliferative control (pCT) group compared with the secretory control (sCT) group. (b) Top 20 enriched GO terms in the BP category among DEmRNAs in the proliferative endometriosis (pEMS) group compared with the secretory endometriosis (sEMS) group. (c) Top 10 enriched KEGG pathways in the pCT group compared with the sCT group. (d) Top 10 enriched KEGG pathways in the pEMS group compared with the sEMS group. The X axis represents the number of genes. **p < 0.001, ***p < 0.0001.
