# Supplementary material for: Expression Profiling of Coding and Noncoding RNAs in the Endometrium of Patients with Endometriosis
Source: Int J Mol Sci. 2024 Oct 1;25(19):10581. doi: 10.3390/ijms251910581 (PMC11476965; doi:10.3390/ijms251910581)
Supplement: Supplementary file 1 [file ijms-25-10581-s001.zip › Figure S2.pptx]

## Slide 1
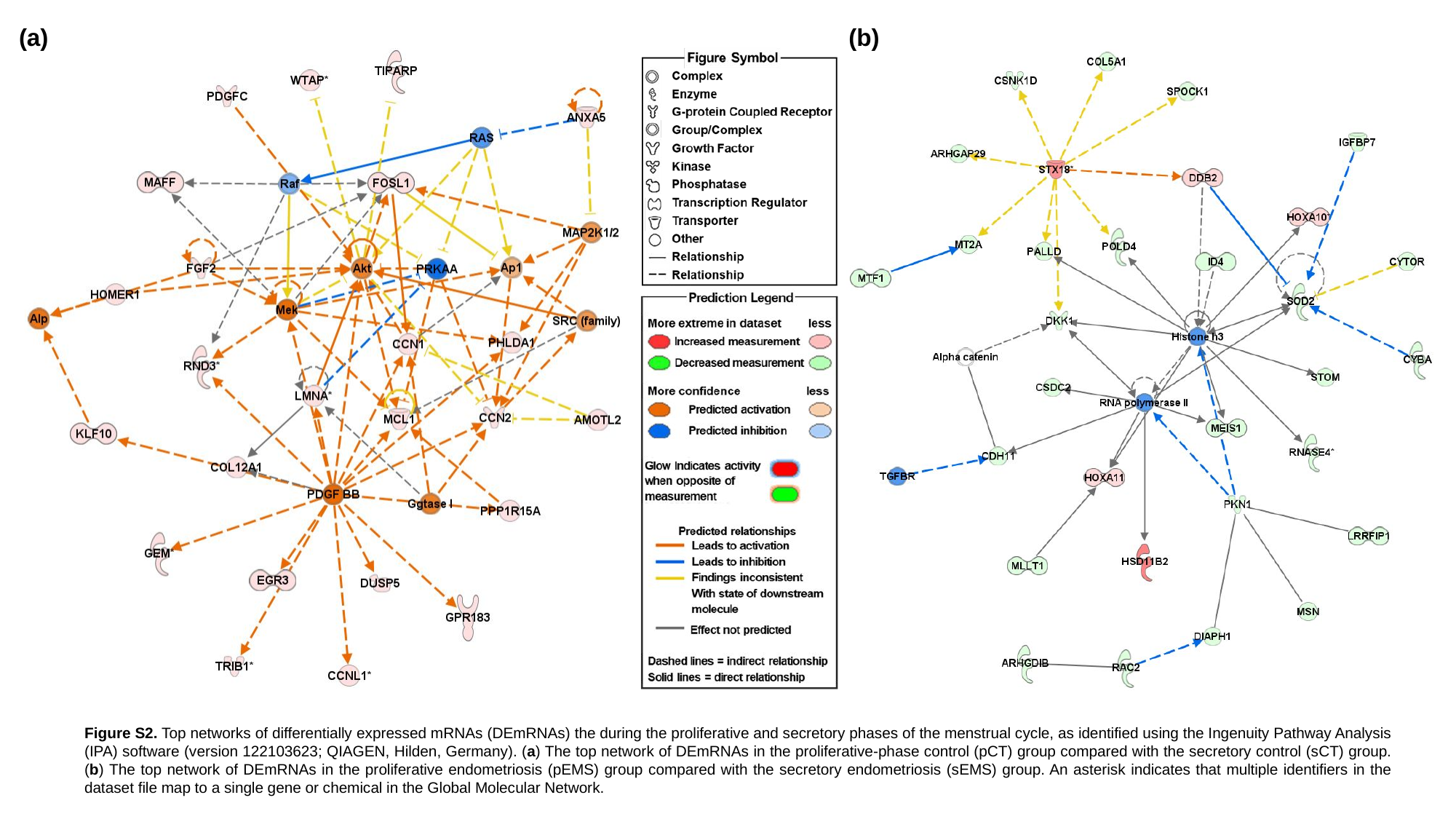

(a)
(b)
Figure S2. Top networks of differentially expressed mRNAs (DEmRNAs) the during the proliferative and secretory phases of the menstrual cycle, as identified using the Ingenuity Pathway Analysis (IPA) software (version 122103623; QIAGEN, Hilden, Germany). (a) The top network of DEmRNAs in the proliferative-phase control (pCT) group compared with the secretory control (sCT) group. (b) The top network of DEmRNAs in the proliferative endometriosis (pEMS) group compared with the secretory endometriosis (sEMS) group. An asterisk indicates that multiple identifiers in the dataset file map to a single gene or chemical in the Global Molecular Network.
