# Supplementary material for: Expression Profiling of Coding and Noncoding RNAs in the Endometrium of Patients with Endometriosis
Source: Int J Mol Sci. 2024 Oct 1;25(19):10581. doi: 10.3390/ijms251910581 (PMC11476965; doi:10.3390/ijms251910581)
Supplement: Supplementary file 1 [file ijms-25-10581-s001.zip › Figure S3.pptx]

## Slide 1
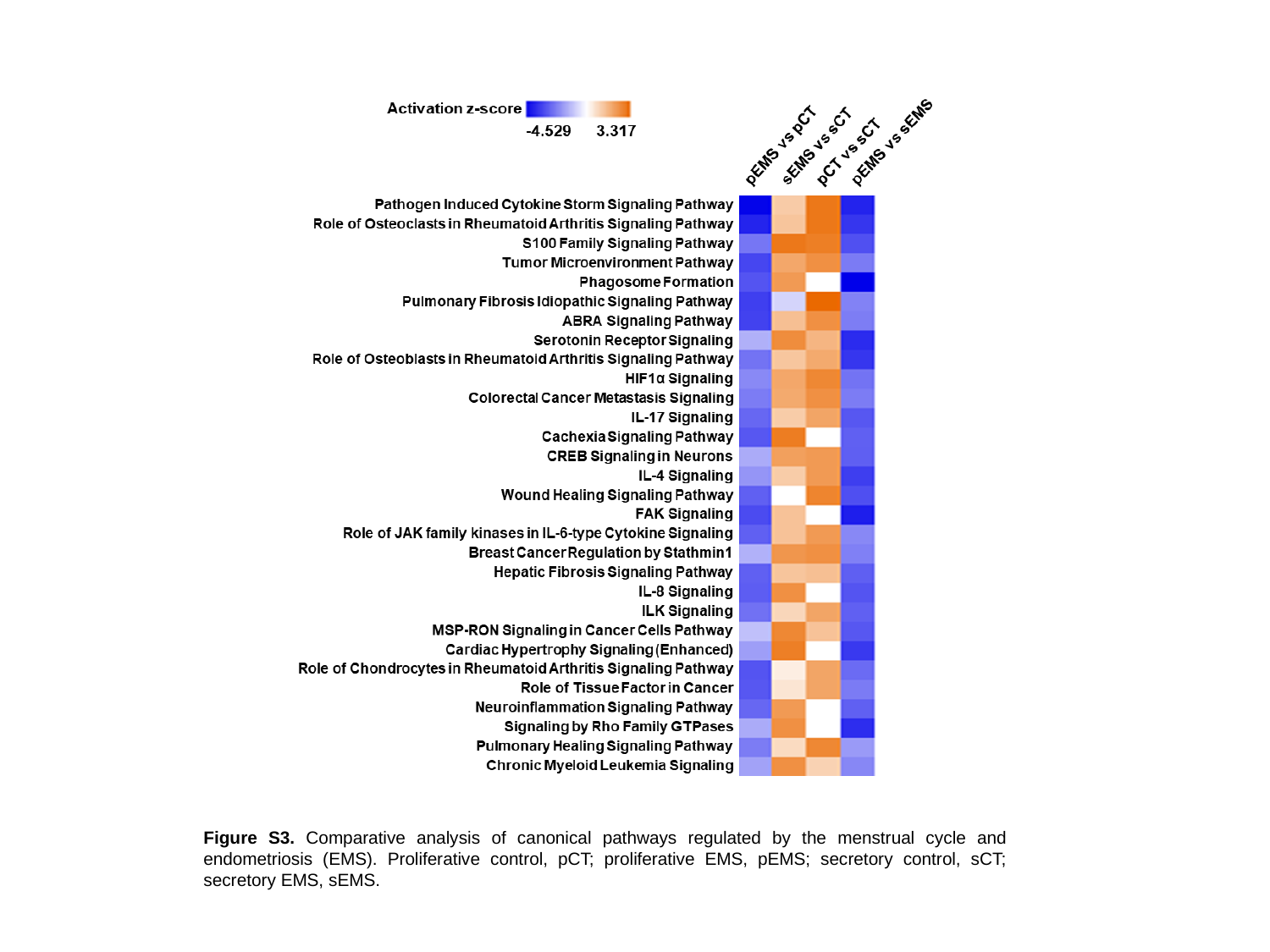

Figure S3. Comparative analysis of canonical pathways regulated by the menstrual cycle and endometriosis (EMS). Proliferative control, pCT; proliferative EMS, pEMS; secretory control, sCT; secretory EMS, sEMS.
