# Supplementary material for: Expression Profiling of Coding and Noncoding RNAs in the Endometrium of Patients with Endometriosis
Source: Int J Mol Sci. 2024 Oct 1;25(19):10581. doi: 10.3390/ijms251910581 (PMC11476965; doi:10.3390/ijms251910581)
Supplement: Supplementary file 1 [file ijms-25-10581-s001.zip › Table S4.pdf]

Table S4. List of mRNAs selected from total RNA-Seq results for validation

Gene symbol	Fold change			
	pEMS vs pCT	sEMS vs sCT	pCT vs sCT	pEMS vs sEMS
GRHL2	2.540	0.731	0.594	2.065
PIK3R1	2.139	1.817	-1.716	-1.458
ELP3	3.036	1.064	-1.447	1.973
SRPX2	-4.041	2.528	3.826	-2.670
DUSP1	-9.398	-1.285	2.727	-2.682
DUSP5	-11.602	-1.181	4.969	-1.977
PLAUR	-4.670	1.427	3.124	-2.133
CREB5	-5.321	-1.455	3.669	1.003
PLK3	-4.189	1.053	3.016	-1.463
NR4A1	-19.617	-2.404	5.199	-1.570
XIAP	1.354	2.590	1.415	-1.352
EDNRB	-1.374	5.407	-1.066	-7.919
LAMB3	-1.337	5.935	-1.241	-9.847
IL6ST	1.111	3.272	-1.489	-4.387
HIF1A	-1.083	2.366	1.283	-1.998
SOD2	-1.475	3.835	-1.094	-6.190
EDNRA	-1.578	-2.011	1.261	1.607
PAM	-1.104	-4.259	1.506	5.808
MAP2K6	3.917	-3.207	-1.950	6.443

EMS, endometriosis; control, CT; proliferative EMS, pEMS; proliferative CT, pCT; secretory EMS, sEMS, secretory CT, sCT.
