# Supplementary material for: Expression Profiling of Coding and Noncoding RNAs in the Endometrium of Patients with Endometriosis
Source: Int J Mol Sci. 2024 Oct 1;25(19):10581. doi: 10.3390/ijms251910581 (PMC11476965; doi:10.3390/ijms251910581)
Supplement: Supplementary file 1 [file ijms-25-10581-s001.zip › Table S5.pdf]

Table S5. Primers used in qRT-PCR

Gene	Forward (5'-3')	Reverse (5'-3')	Amplicon size (bp)
U6	GCTTCGGCAGCACATATACTAAAAT	CGCTTCACGAATTTGCGTGTCAT	89
NONHSAT008415.2	GGAAGGCAAACACATCAATGG	CACCCCTAAAAATTCCCTCATG	80
NONHSAG019547.2	TCCAGGGTAAAGTCAGGCAC	GGGCCACAGCTAAAACCAAA	90
NONHSAG019742.2	GGGAGCTTGGGCTTCATTCT	AGCCCCTTCTGTAGTCCTGT	150
NONHSAT120701.2	AGATGATCTGCACTGTCAAGT	TTCACTGAAGGCTGCCGAAT	231
NONHSAG048398.2	GCTGTCAAGAAGACCCACAC	GCTGGAGACATCTGCATCTGA	128
NONHSAG016560.2	ATCCGATTACCCCACTGCTG	CCGGCACAAAGTTGCAGTTA	108
NONHSAT149106.1	GCTTGAATGACTTTTTGGACCTCT	AACTGTCTGGCTTGTGACCC	162
NONHSAG026040.2	TCCATCACAAAAGCCGAGCC	TTGTTTCGGCACTTTGCAGC	91
NONHSAT000959.2	TCTGGTGGTGAAAGCAGCC	CATTTCTCAGTCCGGCCCC	170
NONHSAT203423.1	CCCTCAAGTGAGTATGTCCGA	AAAGTTCCAGCGGATGTCGT	201
NONHSAG001763.2	CTTGCAGGGGAGGAAGCTAAA	GCACGCTCTTTCAGTCCGT	97
NONHSAG053769.2	TCTTCATCTGGCTGCTGGTG	GTAGTGAGAGCTTGCGGAGG	83
NONHSAG012105.2	TGCCTCTCAAGAGCCACAAA	TCCAGCAGCCAAGATTCAGA	80
NONHSAG020839.2	CTCCCTCCTGCTCACAAAGG	CTGGAGCAAGACAGGGAAGG	213
lnc-CAMKK2-3:2	AGAAAACGGCCATCTGGAGG	TGCTAGCTCCTGCTTCTCTC	91
NONHSAG039821.2	CTACTGCTGTCCACCCACAC	CAGCAGGTAGGCATTGGGAA	132
GAPDH	ACCCAGAAGACTGTGGATGG	TTCTAGACGGCAGGTCAGGT	201
GRHL2	TCAATACCCGAAGAGCCTACA	CTTGGCTGTCACTTGCTTTGC	201
PIK3R1	AAGAAGTTGAACGAGTGGTTGG	GCCCTGTTTACTGCTCTCCC	192
ELP3	CTGCCGTCCCTCCTCAGTAT	ACAGCCACGACAGCAATCC	91
SRPX2	CCACATGCTACTCACCGAAGG	GTAGTGCGTGGCATCTCATCT	171
DUSP1	GCCTTGCTTACCTTATGAGGAC	GGGAGAGATGATGCTTCGCC	86
DUSP5	TGTCGTCCTCACCTCGCTA	GGGCTCTCTCACTCTCAATCTTC	152
PLAUR	TGTAAGACCAACGGGGATTGC	AGCCAGTCCGATAGCTCAGG	166
CREB5	CCCTGCCCAACCCTACAATG	GGACCTTGCATCCCCATGAT	97
PLK3	AGCGCCTACGCTGTCAAAG	CTCAAAGTGGTGCGAAAAACG	135
NR4A1	ATGCCCTGTATCCAAGCCC	GTGTAGCCGTCCATGAAGGT	182
XIAP	ACCGTGCGGTGCTTTAGTT	TGCGTGGCACTATTTTCAAGATA	134
EDNRB	GTCCCAATATCTTGATCGCCAG	AAGGCACCAGCTTACACATCT	132
LAMB3	CCAAAGGTGCGACTGCAATG	AGTTCTTGCCTTCGGTGTGG	125
IL6ST	ACCATCCCACTCACACCTCA	ACCATCCCACTCACACCTCA	121

HIF1A	GAACGTCGAAAAGAAAAGTCTCG	CCTTATCAAGATGCGAACTCACA	124
SOD2	GGACACTTACAAATTGCTGCTTGT	AGTAAGCGTGCTCCCACACAT	100
EDNRA	TCGGGTTCTATTTCTGTATGCCC	TGTTTTTGCCACTTCTCGACG	143
PAM	CTGGGGTTACACCTAAACAGTC	GCTTGAAGTCAATCACGAAGGC	90
MAP2K6	GAAGCATTTGAACAACCTCAGAC	CCTGGCTATTTACTGTGGCTC	223
PALLD	GGAACGAAAACTTCGCTTCAAG	CACTGTCAGCACCTAGCAGTC	101
THBS1	AGACTCCGCATCGCAAAGG	TCACCACGTTGTTGTCAAGGG	157
VEGFA	AGGGCAGAATCATCACGAAGT	AGGGTCTCGATTGGATGGCA	75
DHRS3	TTCCTGCCACGTATGCTGG	TTTGGATGTGCAGTAGTCGATG	105
IGF1	GCTCTTCAGTTCGTGTGTGGA	GCCTCCTTAGATCACAGCTCC	133
